# Devemos Considerar a Estimulação da Guanilil Ciclase Solúvel como Benéfica para o Tratamento da Hipertensão Pulmonar Pré-Capilar?

**DOI:** 10.36660/abc.20220261

**Published:** 2022-06-06

**Authors:** Allan Kardec Nogueira de Alencar

**Affiliations:** 1 Faculdade de Medicina de Petrópolis Petrópolis RJ Brasil Faculdade de Medicina de Petrópolis, Petrópolis, RJ – Brasil

**Keywords:** Hipertensão Pulmonar, Circulação Pulmonar, Pressão Arterial Pulmonar de Oclusão, Estimuladores da Guanilil Ciclcase Solúvel, Riociguate

Um dos mais raros e complexos grupos de doenças que acometem o sistema cardiopulmonar é conhecido como hipertensão pulmonar (HP), uma condição clínica com risco de vida que em estágios avançados eventualmente resulta em disfunção irreversível da câmara cardíaca direita e morte súbita cardíaca.^1^ A hipertensão arterial pulmonar (HAP) e a HP tromboembólica crônica (HPTEC) são dois grupos distintos dentro do sistema de classificação clínica da HP, em que a perda e o remodelamento obstrutivo dos vasos pulmonares são responsáveis por um aumento significativo da pressão arterial pulmonar (PAP) e da resistência vascular pulmonar (RVP), resultando em declínio funcional do desempenho cardíaco e insuficiência progressiva do ventrículo direito (VD).^1^

A HAP é uma HP do tipo pré-capilar (Grupo 1), definida hemodinamicamente por uma pressão arterial pulmonar de oclusão média (mPAP) > 20 mmHg, pressão arterial pulmonar de oclusão PA ≤ 15 mmHg e PVR ≥ 3 unidades Wood.^2^ O remodelamento dos vasos pulmonares na HAP é representado, nas paredes das artérias pulmonares (APs), pelo acúmulo de células musculares lisas (CMLAPs) e endoteliais (CEAPs), fibroblastos, miofibroblastos e pericitos. Além disso, esse processo de remodelamento resulta em perda de artérias pré-capilares e exacerba a inflamação perivascular.^1^ A perda excessiva de (CEAPs) é uma característica patológica chave da HAP.^3^ Este fenômeno desencadeia o desenvolvimento de um fenótipo hiperproliferativo e resistente à apoptose das CEAPs.^3^ Subsequentemente, uma intensa proliferação de CEAPs induz a formação de lesões plexogênicas nos vasos pulmonares, uma característica histopatológica da HAP.^4^

Pacientes com doença tromboembólica podem, consequentemente, desenvolver HPTEC (Grupo 4 na classificação HP)^2^ devido a uma obstrução vascular pulmonar persistente após um evento embólico.^2^ Fisiopatologicamente, a HPTEC pode ser multifatorial, pois envolve tanto os grandes vasos pulmonares quanto a microcirculação.^5^ Setenta e cinco por cento dos pacientes com HP em doença tromboembólica crônica têm história de embolia pulmonar aguda,^6^ e foi sugerido que os 25% restantes apresentavam êmbolos recorrentes e silenciosos.^6^ Ressaltando as características histopatológicas da HPTEC, principalmente materiais trombóticos com grande quantidade de colágeno, elastina, raras calcificações, e comumente células inflamatórias aderem às paredes dos vasos pulmonares e obliteram esse pequeno leito vascular.^7^ Assim como a HAP, a HPTEC é outro exemplo de HP pré-capilar, na qual os pacientes podem ser diagnosticados hemodinamicamente com pPAP de oclusão ≤ 15 mmHg, RVP ≥ 3 unidades Wood e mPAP variando de 15 a 24 mmHg.^2^

Os tratamentos disponíveis para HP visam especificamente a redução da vasoconstrição de APs e da sobrecarga de pressão sobre o ventrículo direito. (VD).^1,8,9^ Foi relatado que a estimulação da enzima guanilil ciclase solúvel (GCs) com um fármaco denominado riociguate é benéfica no quadro clínico da HAP.^10^ No contexto da HPTEC, a endarterectomia pulmonar é o tratamento recomendado.^11^ No entanto, até 40% dos pacientes são tecnicamente inoperáveis e 17-31% desenvolvem HP persistente ou recorrente após a endarterectomia pulmonar.^11^ É importante ressaltar que o riociguate foi a primeira substância a ser aprovada para o tratamento de dois grupos distintos de HP pré-capilar: HAP e HPTEC inoperável ou persistente/recorrente.^11^

Molecularmente, em CMLAPs de pacientes com HAP e HPTEC, o eixo óxido nítrico (NO)-sGC-GMP cíclico (cGMP) está desregulado, o que resulta em inflamação vascular pulmonar, trombose e vasoconstrição exacerbada.^1,4,5^ O riociguate modifica a via de sinalização do GMPc ao aumentar os níveis citosólicos desse nucleotídeo cíclico, após estímulo da GCs. Deve-se notar que esse mecanismo é independente da ativiade parácrina do NO nas células vasculares pulmonares.^12^ Níveis citosólicos aumentados de cGMP levam à vasodilatação e inibição da proliferação e fibrose de CMLAPs, com efeitos antitrombóticos e anti-inflamatórios adicionais.^12^ Além disso, o aumento do conteúdo de GMPc após a administração de riociguate pode levar à inibição da fosfodiesterase tipo 3 em cardiomiócitos, o que consequentemente aumenta os níveis intracelulares de AMP cíclico e promove um efeito inotrópico positivo no coração.^12^ O riociguate também pode exercer efeitos cardioprotetores e melhorar a função do VD quando potencializa a ativação da proteína cinase G, acompanhando o aumento dos níveis de cGMP.^12^ Essa sinalização biomolecular é explicada principalmente pela abertura dos canais K_ATP_ mitocondriais nas células cardíacas.^12^

Em seu artigo inovador de 2022, Spilimbergo et al.,^13^ foram os primeiros pesquisadores a investigar retrospectivamente os efeitos do riociguate em pacientes com HAP e HPTEC por meio de um estudo real de acompanhamento de 3 anos.^13^ Esses cientistas mediram os parâmetros atuais de avaliação de risco e descobriram dados interessantes que podem ajudar a comprovar os efeitos benéficos do riociguate em indivíduos com HAP e HPTEC.^13^

Em primeiro lugar, eles mostraram que o riociguat aumentou significativamente a distância percorrida em 6 minutos (DTC6) após pelo menos 3 anos de terapia, em comparação com os dados basais, em pacientes com HAP e HPTEC.^13^ Os autores também encontraram um aumento gradual da DTC6 de 3 meses a 3 anos após o início do tratamento de pacientes com riociguate, com mediana final superior a 440 metros.^13^

É importante ressaltar que, após 3 anos de investigação, os autores não observaram alterações significativas nos seguintes parâmetros: PAP sistólica, PAP diastólica, PAPm, RVP, índice cardíaco, débito cardíaco e BNP do pró-hormônio N-terminal (NT) (NT-pro BNP).^13^ No entanto, 3 anos de tratamento com riociguate aumentaram significativamente a PAP.^13^

De acordo com os achados citados acima, os autores demonstraram que a estimulação da GCs nesta coorte diminuiu o número de pacientes em classe funcional III da Organização Mundial da Saúde (OMS), que foram então classificados como classe funcional II após o acompanhamento.^13^ Considerando apenas os pacientes que completaram 3 anos de acompanhamento, no início do estudo, 61% dos pacientes estavam em classe funcional III, e após 3 anos de tratamento com riociguate, 10% dos pacientes continuaram em classe funcional III.^13^ Da mesma forma, no início do estudo, 32% dos pacientes estavam em classe funcional II e, após o tratamento, 71% dos pacientes estavam em classe funcional II.^13^ Também foi demonstrado que a taxa de sobrevida em três anos entre os pacientes com HAP e HPTEC tratados com riociguate foi de 96,7%.^13^ Portanto, podemos entender que o riociguate melhorou a capacidade funcional de exercício, aumentou a PAP de oclusão e preservou as demais medidas clínicas e laboratoriais após 3 anos de tratamento, o que provavelmente transferiu a maioria dos pacientes para uma melhor classe funcional da OMS.

Finalmente, de acordo com a estratificação de risco não invasiva francesa, os pesquisadores descobriram que nenhum paciente estava em baixo risco no início do estudo, mas 7 pacientes alcançaram o status de baixo risco após 3 anos de terapia com riociguate.^13^

Na minha opinião, os autores conduziram esta investigação de forma adequada e mostraram as limitações do estudo na seção de discussão. Nesse sentido, este trabalho pode agregar dados importantes sobre a terapêutica da HP pré-capilar, embora ainda entendamos que há carência de agentes pleiotrópicos no contexto dessas doenças, principalmente quando destacamos a necessidade de novas abordagens farmacológicas que promovam ações benéficas no leito vascular pulmonar (atenuação do fenótipo proliferativo de células endoteliais, musculares lisas e fibroblastos) com potencial efeito cardioprotetor adicional.
