# Supplementary material for: Devemos Considerar a Estimulação da Guanilil Ciclase Solúvel como Benéfica para o Tratamento da Hipertensão Pulmonar Pré-Capilar?
Source: Arq Bras Cardiol. 2022 Jun 6;118(6):1067–8. [Article in Portuguese] doi: 10.36660/abc.20220261 (PMC9345151; doi:10.36660/abc.20220261)
Supplement: Translation

# Translation: Should we Consider the Stimulation of Soluble Guanylyl Cyclase as Beneficial for Treating Pre-Capillary Pulmonary Hypertension?

**Authors:** Allan Kardec Nogueira de Alencar

**Affiliations:** 1 Petrópolis RJ Brazil Faculdade de Medicina de Petrópolis, Petrópolis, RJ – Brazil

**Keywords:** Pulmonary hypertension, Pulmonary Ciruculation, Pulmonary Arterial Wedge Pressure, Soluble Guanylyl Cyclase Stimulators, Riociguat

One of the rarest and most complex group of diseases that affects the cardiopulmonary system is known as pulmonary hypertension (PH), a life-threatening clinical condition that in advanced stages eventually results in irreversible dysfunction of the right heart chamber and sudden cardiac death.^1^ Pulmonary arterial hypertension (PAH) and chronic thromboembolic PH (CTEPH) are two different groups within the PH clinical classification system, in which loss and obstructive remodeling of the lung vessels is responsible for a significant rise in pulmonary arterial (PA) pressure and pulmonary vascular resistance (PVR), resulting in a functional decline of the heart performance and progressive right ventricle (RV) failure.^1^

PAH is a pre-capillary-type PH (Group 1), hemodynamically defined by a mean pulmonary arterial pressure (mPAP) >20 mmHg, PA wedge pressure ≤15 mmHg, and PVR ≥3 Wood units.^2^ Remodeling of pulmonary vessels in PAH is depicted by the accumulation of pulmonary artery smooth muscle (PASMCs) and endothelial cells (PAECs), fibroblasts, myofibroblasts, and pericytes in the PA walls. In addition, this remodeling process results in a loss of pre-capillary arteries and exacerbates perivascular inflammation.^1^ The excessive loss of PAECs is a key pathobiological feature of PAH.^3^ This phenomenon triggers the development of an apoptosis-resistant and hyperproliferative phenotype of PAECs.^3^ Subsequently, an intense proliferation of PAECs induces the formation of plexogenic lesions in the lung vessels, a histopathologic hallmark of PAH.^4^

Patients with thromboembolic disease may consequently develop CTEPH (Group 4 in the PH classification)^2^ due to a persistent pulmonary vascular obstruction after an embolic event.^2^ Pathophysiologically, CTEPH can be multifactorial as it involves both large pulmonary vessels and microcirculation.^5^ 75% of patients with PH in chronic thromboembolic disease have a history of acute pulmonary embolism,^6^ and it was suggested that the remaining 25% had recurrent and silent emboli.^6^ Pointing out the histopathological characteristics of CTEPH, mainly thrombotic materials with a large amount of collagen, elastin, rarely calcifications, and commonly inflammatory cells adhere to the pulmonary vessel walls and obliterate this small vascular bed.^7^ Similarly to PAH, CTEPH is another example of pre-capillary PH, in which patients can be hemodynamically diagnosed with a pulmonary arterial wedge pressure ≤15 mmHg, PVR ≥3 Wood units and mPAP ranging from 15 to 24 mmHg.^2^

Available treatments for PH specifically target the reduction of PA vasoconstriction and the pressure-overloaded RV.^1,8,9^ It was reported that stimulation of the soluble guanylyl cyclase (sGC) enzyme with a drug named riociguat is beneficial in the clinical setting of PAH.^10^ In the context of CTEPH, pulmonary endarterectomy is the recommended treatment.^11^ However, up to 40% of patients are technically inoperable, and 17-31% develop persistent or recurrent PH following the pulmonary endarterectomy.^11^ Importantly, riociguat was the first substance to be approved for the treatment of two distinct groups of pre-capillary PH: PAH and inoperable or persistent/recurrent CTEPH.^11^

Molecularly, in PASMCs from patients with PAH and CTEPH, the nitric oxide (NO)-sGC-cyclic GMP (cGMP) axis is deregulated, which results in pulmonary vascular inflammation, thrombosis and exacerbated vasoconstriction.^1,4,5^ Riociguat modifies the cGMP signaling pathway by increasing its cytosolic levels after stimulation of sGC. It should be addressed that this mechanism is independent of the paracrine roles of NO in the pulmonary vascular cells.^12^ Increased cytosolic levels of cGMP lead to vasodilation and inhibition of PASMCs proliferation and fibrosis, with further antithrombotic and anti-inflammatory effects.^12^ Additionally, the increasing content of cGMP after administration of riociguat could lead to inhibition of the phosphodiesterase type 3 in cardiomyocytes, which consequently augments the intracellular levels of cyclic AMP and promotes a positive inotropic effect in the heart.^12^ Riociguat may also exert cardioprotective effects and improve the RV function when it potentiates the activation of protein kinase G, following the rise of cGMP levels.^12^ This biomolecular signaling is mainly explained by the opening of mitochondrial K_ATP_ channels in cardiac cells.^12^

In their groundbreaking paper of 2022, Spilimbergo et al.^13^ were the first researchers to retrospectively investigate the effects of riociguat in patients with PAH and CTEPH through a 3-year follow-up real-life study.^13^ These scientists measured current risk assessment parameters and found interesting data which may help to prove the beneficial effects of riociguat in PAH and CTEPH subjects.^13^

Firstly, they have shown that riociguat significantly increased the 6-minute walking distance (6MWD) after at least 3 years of therapy, compared with the baseline data, in both patients with PAH and CTEPH.^13^ The authors also found a gradual increase in the 6MWD from 3 months to 3 years after the beginning of the treatment of diseased subjects with riociguat, with a final median greater than 440 meters.^13^

Importantly, after 3 years of investigation, the authors did not observe significant changes in the following parameters: systolic pulmonary arterial pressure, diastolic pulmonary arterial pressure, mPAP, PVR, cardiac index, cardiac output and N-terminal (NT)-prohormone BNP (NT-pro BNP) levels.^13^ However, 3 years of treatment with riociguat significantly increased the pulmonary arterial wedge pressure.^13^

In accordance with the findings mentioned above, the authors have shown that the stimulation of sGC in this cohort decreased the number of patients in the World Health Organization (WHO) functional class III, who were then classified as functional class II after the follow-up.^13^ Considering only the patients who completed 3 years of follow-up, at baseline, 61% of patients were functional class III, and after 3 years of treatment with riociguat, 10% of patients continued as functional class III.^13^ Similarly, at baseline, 32% of the patients were in functional class II, and after treatment, 71% of the patients were in functional class II.^13^ It was also shown that the three-year survival rate among PAH and CTEPH patients treated with riociguat was 96.7%.^13^ Therefore, we might understand that riociguat has improved the functional exercise capacity, increased the pulmonary arterial wedge pressure and preserved the other clinical and laboratorial measurements after 3 years of treatment, which probably have transferred most patients to a better WHO functional class.

Finally, according to the French non-invasive risk stratification, the researchers found that no patient was at low risk at baseline, but 7 patients achieved low-risk status after 3 years of therapy with riociguat.^13^

In my opinion, the authors have conducted this investigation appropriately and have shown the study’s limitations in the discussion section. Accordingly, this work can add important data on the therapy for pre-capillary PH, although we still understand that there is a lack of pleiotropic agents in the context of these diseases, mainly when we highlight the need for new pharmacological approaches that promote beneficial actions on the pulmonary vascular bed (attenuation of the proliferative phenotype of endothelial, smooth muscle and fibroblast cells) with a further potential cardioprotective effect.
